# Pleiotropic Roles of Non-Coding RNAs in TGF-β-Mediated Epithelial-Mesenchymal Transition and Their Functions in Tumor Progression

**DOI:** 10.3390/cancers9070075

**Published:** 2017-07-01

**Authors:** Simon Grelet, Ariel McShane, Renaud Geslain, Philip H. Howe

**Affiliations:** 1 Department of Biochemistry and Molecular Biology, MUSC, Charleston, SC 29425, USA; grelet@musc.edu; 2 Laboratory of tRNA Biology, Department of Biology, College of Charleston, Charleston, SC 29424, USA; mcshaneab@g.cofc.edu

**Keywords:** epithelial-mesenchymal transition, tumor progression, metastasis, TGF-β, non-coding RNA, tRNA, post-transcriptional regulation

## Abstract

Epithelial-mesenchymal transition (EMT) is a spatially- and temporally-regulated process involved in physiological and pathological transformations, such as embryonic development and tumor progression. While the role of TGF-β as an EMT-inducer has been extensively documented, the molecular mechanisms regulating this transition and their implications in tumor metastasis are still subjects of intensive debates and investigations. TGF-β regulates EMT through both transcriptional and post-transcriptional mechanisms, and recent advances underline the critical roles of non-coding RNAs in these processes. Although microRNAs and lncRNAs have been clearly identified as effectors of TGF-β-mediated EMT, the contributions of other atypical non-coding RNA species, such as piRNAs, snRNAs, snoRNAs, circRNAs, and even housekeeping tRNAs, have only been suggested and remain largely elusive. This review discusses the current literature including the most recent reports emphasizing the regulatory functions of non-coding RNA in TGF-β-mediated EMT, provides original experimental evidence, and advocates in general for a broader approach in the quest of new regulatory RNAs.

## 1. Introduction

Metastasis represents a critical step in tumor progression that is responsible for more than 90% of cancer-induced mortality. Despite tremendous efforts from the scientific community, the cellular and molecular events that specifically control metastatic colonization are still poorly understood.

**Figure 1:**
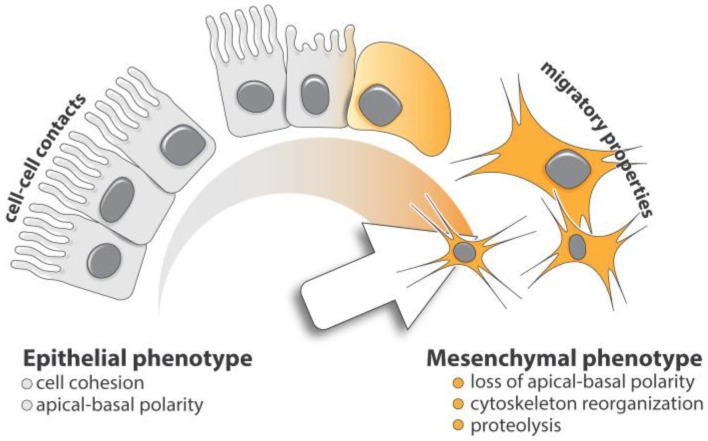
Cell plasticity in EMT. Epithelial-mesenchymal transition is a multistep process allowing epithelial cells to acquire mesenchymal phenotype. Upon TGF-β exposure, epithelial cells lose their apical-basal polarity and cellular junctions leading to a loss of cell-cell cohesion. Through a complex and regimented cellular and molecular program, these cells progressively gain mesenchymal features, including cytoskeleton reorganization and proteolytic capacity favoring efficient cell motility.

Epithelial-mesenchymal transition (EMT) is key in both embryonic development and tumor metastasis. EMT consists of a fine-tuned phenotypic switch characterized by the loss of apical-basal polarity and cellular adhesion in epithelial cells [1,2]. Cells undergoing transition gradually express mesenchymal features, such as enhanced cytoskeletal rearrangement and extracellular matrix (ECM) degradation, both essential for cell motility (Figure 1). While the role of EMT in metastasis progression is still debated, its implication in the increased resistance seen in both conventional and targeted antitumor therapies is well established [1,3,4,5,6,7,8].

The transcriptional mechanisms controlling EMT are particularly well documented, however, evidences of post-transcriptional regulation are now emerging in the literature, urging the scientific community to consider and investigate the synergistic combinations of these two levels of controls. At the cellular level, the tumor microenvironment (TME), including cancer associated fibroblasts (CAF), immune and endothelial cells, as well as the extracellular matrix (ECM) composition, are contributing factors modulating EMT and metastasis [7,9,10]. In addition to its structuring role, the ECM contains numerous cytokines, such as TGF-β. TGF-β signaling has a predominant function in suppressing the growth of normal epithelial cells. It also drives the metastatic process in malignantly-transformed tumor cells. Other growth factors, such as epidermal growth factor (EGF), fibroblast growth factor (FGF), and vascular endothelial growth factor (VEGF) were also clearly identified to be involved in EMT [11].

More recently, the development and improvement of transcriptomics boosted the discovery of new non-coding RNAs harboring regulatory functions. These species include PIWI-interacting RNA (piRNAs), small nuclear RNA (snRNAs), small nucleolar RNA (snoRNAs), circular RNAs (circRNAs), transfer RNAs (tRNAs), microRNAs (miRNAs), and long non-coding RNAs (lncRNAs). These RNA species operate through various molecular mechanisms including transcriptional and post-transcriptional controls.

In this article, we discuss the recent advances on the role of non-coding RNA in the regulation of TGF-β-induced EMT during tumor progression. We also present and comment on unpublished data collected in our laboratory regarding the regulation of tRNA expression in an in vitro model of TGF-β-induced-EMT of human tumor cells.

## 2. Cellular Basis of TGF-β-Induced EMT

**Figure 2:**
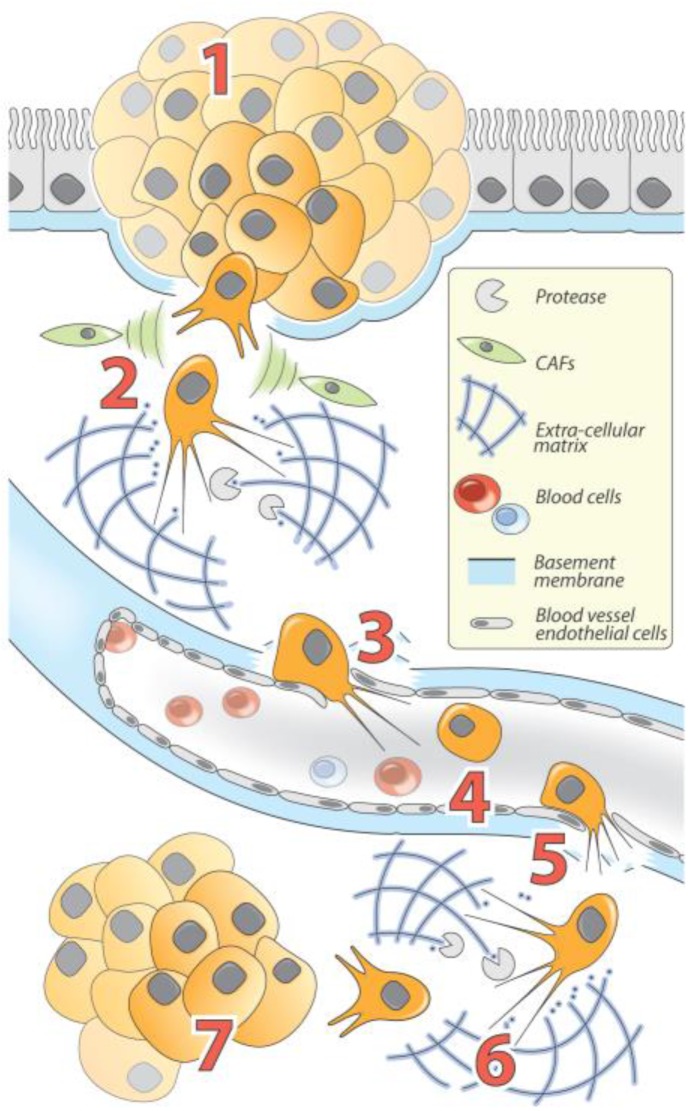
The Role of EMT and MET in carcinomas progression. (**1**) Following carcinogenesis, epithelial tumor cells proliferate to develop primary tumors called carcinoma in situ. In response to acquired mutations and/or exogenous stimuli, tumor cells gain invasive properties allowing them to break the basement membrane. Tumor cells then (**2**) invade and spread to surrounding tissues and structures and interact with numerous TME factors including cytokine-secreting CAFs, which reinforce EMT and invasion processes (cytoskeleton reorganization and increased proteolytic activity allow cells to degrade and invade the extracellular matrix (ECM)); (**3**) penetrate the vascular system (intravasation); (**4**) circulate throughout the body; (**5**) leave the vascular system to invade distant tissues (extravasation); (**6**) colonize distant sites through ECM degradation and invasion; and (**7**) reacquire epithelial phenotypes through MET and proliferate to ultimately form a metastasis.

The TGF-β signaling pathway was initially described for its critical role in cell proliferation and EMT during embryonic development of the neural crest, the somites, the heart, and various craniofacial structures [12]. TGF-β-induced EMT also manifests in pathological contexts in adults, specifically, during cancer progression and fibrosis. Due to its transient and reversible nature, EMT is technically challenging to observe throughout tumor progression in vivo. Nevertheless, it was proposed that during cancer progression of epithelial tumors, cells become significantly more invasive after completing EMT. In the current model, EMT-positive tumor cells, displaying newly-acquired mesenchymal features, are capable of invading their surrounding environment and complete extravasation in the circulatory system, resulting in ECM degradation and increased motility. It was proposed that the changes in cell plasticity induced by EMT enhance the ability of the circulating tumor cells (CTCs) to survive in the blood stream [6,13]. CTCs then re-invade distant organs through the extravasation process. Finally, metastatic colonization is initiated by the re-epithelialization of the cells through the reverse mechanism, called MET (mesenchymal-epithelial transition), followed by either a proliferative or dormancy step, which are responsible for secondary tumor growth and drug resistance or later tumor relapse, respectively (Figure 2).

## 3. Molecular Mechanisms of TGF-β-Induced EMT

### 3.1. Transcriptional Regulation of TGF-β-Induced EMT in Tumor Cells

EMT-inducing signals are cell- or tissue-specific and require the cooperation of multiple signaling pathways involving numerous regulators. TGF-β is arguably the most powerful EMT inducer in tumor cells, as it coordinates EMT at several levels and its impact on transcription has now been well documented. The TGF-β pathway is initiated by a superfamily of TGF-β ligands, including three forms of TGF-β ligands (TGF-β1 to -β3) and BMP isoforms (BMP2 to -7), whose secretion depends on tumor context and TME. TGF-β-mediated EMT integrates both Smad and non-Smad signaling pathways and is usually characterized by a loss of epithelial cell markers, such as E-Cadherin, and tight junction proteins in addition to the expression of mesenchymal cell markers, such as N-cadherin and vimentin. The epithelial-to-mesenchymal switch is triggered by a tightly-regulated transcription program that involves EMT-inducing transcription factors (EMT-TFs). These specific transcription factors are themselves activated by Smad signaling or other pathways, such as ErK/MAPK, RhoGTPases and PI3K/Akt, that also contribute to tumor progression through regulation of cytoskeleton organization, cell growth, survival, migration, and invasion [14,15]. Effectors of TGF-β signaling include the Krüppel-like factor 8 (KLF8) [16], the Brachyury factor [17], Goosecoid, TCF4, PRRX1 [18], basic helix-loop-helix factors (Twist and E12/E47), the Snail family of zinc-finger transcription factors (Snail, Slug, and Smuc) [19,20], and the δEF1 family of two-handed zinc-finger factors (δEF1/ZEB1 and Sip1/ZEB2) [2]. With the exception of Twist, Goosecoid and PRRX1, all these effectors directly repress the transcription of the epithelial marker E-Cadherin (CDH1) by binding to the corresponding gene promoter. Conversely, transcriptional activators of E-Cadherin such as Grhl2 or Elf5 inhibit the TGF-β induced EMT in tumor cells [21,22].

Finally, a growing body of evidence suggests that epigenetic modifications support an additional level of transcriptional control. Dynamic changes in the DNA methylome [23], histone modifications, as well as the differential expression of numerous histone modification factors [24,25], were identified as modulators of TGF-β-induced EMT and metastasis. During metastatic colonization, these epigenetic alterations established during EMT are reversed to promote MET [26,27].

### 3.2. Post-Transcriptional Regulation of TGF-β-Induced EMT in Tumor Cells

Recent data suggest that post-transcriptional regulation of gene expression complements transcriptional regulation during EMT [28,29,30,31]. Several RNA binding proteins (RBPs) directly control the translation of EMT-related genes. For instance, the Nanos homolog 3 RBP (Nanos3, Nos3) translationally controls EMT and metastasis in non-small cell lung cancer (NSCLC) cells by binding to the vimentin mRNA and increasing the expression of mesenchymal marker [31]. In addition, overexpression of YB-1 in breast epithelial cells triggers EMT and enhances metastatic potential by directly activating the cap-independent translation of mRNA encoding transcription factors implicated in EMT, such as Snail1 [28]. Finally, the protein hnRNP E1 (PCBP1) silences the translation of a cohort of mesenchymal mRNAs by directly binding to their targets and inhibiting translation at the elongation step [29,30,32].

## 4. Role of Non-Coding RNAs in TGF-β-Induced EMT

### 4.1. miRNAs

MicroRNAs are small noncoding RNA that modulate a wide range of biological processes including cell differentiation, proliferation, migration, invasion, and cell death. They silence mRNA translation by binding to the 3′-UTRs of target mRNAs resulting in translation inhibition or mRNA degradation. miRNAs are generated as pri-miRNA precursors and then processed into one or multiple mature miRNAs. miRNAs hybridize their mRNA targets using a complementary 7-nucleotides seed-sequence located at their 5′-end [33]. More than 2000 microRNAs have been characterized in humans. This list was considerably extended by the recent identification of more than 3000 additional miRNAs [34].

Many miRNAs, such as the miR-200 family (miR-200f), miR-205, miR-1, and miR-203, are key effectors of the TGF-β-induced EMT (Table 1). In general, TGF-β downregulates the expression of miRNAs in charge of inhibiting the expression of mesenchymal markers in epithelial cells. Conversely, emerging evidence indicates that several miRNAs regulate TGF-β signaling by targeting various members of its canonical or non-canonical pathways.

The miR-200 family is a group of highly influential miRNAs implicated in the regulation of TGF-β-induced EMT. It consists of five members, organized into two chromosomal clusters, which are expressed as polycistronic transcripts (MiR-200b, miR-200a, and miR-429 on human chromosome 1 and miR-200c and miR-141 on chromosome 12). Although miR-205 does not belong to the miR-200 family, it recognizes common targets and, therefore, complements the role of the miR-200f. Numerous studies demonstrated that TGF-β signaling downregulates the expression or the bioavailability of miR-200f and miR-205 [35]. These miRNAs, expressed by epithelial cells, are known to directly repress both ZEB1 and ZEB2 [35,36] therefore inhibiting the progression of EMT by establishing and maintaining an epithelial phenotype [35]. Interestingly, ZEB proteins and miR200f are reciprocally linked in a feedback loop, each controlling the expression of the other [37]. ZEB factors transcriptionally repress miR-200f by binding to highly-conserved recognition sequences on their promoters, while miR-200f inhibits the expression of ZEBs at the post-transcriptional level by binding to target sequences embedded in their 3′-UTRs. It was, therefore, proposed that the ZEB/miR-200 feedback loop acts as the molecular trigger controlling cellular plasticity in development and disease, and constitutes a significant driving force for cancer progression [5,37].

Although precise alterations of the ZEB/miR-200 balance are able to switch breast cancer cells back and forth between epithelial and mesenchymal states, the induction and maintenance of a stable mesenchymal phenotype requires the establishment of autocrine TGF-β signaling that supports sustained ZEB expression [38]. In addition, the recruitment of histone-modifying complexes by ZEB proteins negatively modulates miR-200f expression through epigenetic modification of the miR-200 loci and therefore amplifies the response to TGF-β [38].

Other miRNAs are known for their contribution in TGF-β-induced EMT. For instance, miR-203 and SLUG (SNAI2) operate as a double negative feedback loop mutually inhibiting their expression and thereby controlling EMT and metastasis [39,40]. Finally, miR-1 and miR-200 were identified as being directly repressed by SLUG during EMT [41].

### 4.2. Long Non-Coding RNAs

LncRNAs are transcripts greater than 200 nucleotides deprived of any protein coding sequences. They are divided into five broad categories, including sense, antisense, bidirectional, intronic, and intergenic, with respect to the nearest protein-coding transcripts [42]. LncRNAs modulate gene expression trough different cis- or trans-acting mechanisms. For instance, competing endogenous RNAs (ceRNAs) serve as molecular decoys for specific miRNAs, decreasing their bioavailability and, therefore, protecting the corresponding target mRNAs [43]. In synergy with miRNAs, they regulate gene expression by modulating transcription, RNA processing, chromatin remodeling, genomic imprinting, and association with proteins. Interestingly, dysregulation of lncRNA expression is frequently observed in human cancers. Since LncRNAs control mechanisms of many cellular processes, they represent appealing targets for the development of anti-tumor therapies.

#### 4.2.1. LncRNA-ATB

TGF-β-induced lncRNA-ATB competitively binds to miR-200f, favoring the expression of ZEB1 and ZEB2 proteins, therefore promoting EMT and invasion in hepatocellular carcinoma cells [44]. In addition, lncRNA-ATB promotes organ colonization of disseminated hepatocellular carcinoma cells by binding to IL-11 mRNA and subsequently activating IL-11/STAT3 signaling. Aberrant lncRNA-ATB expression is observed in various cancer types, such as prostate carcinoma [45], colorectal cancer [46], NSCLC [47], and breast cancer [48] (Table 1).

#### 4.2.2. MALAT1

Induction of the metastasis-associated lung adenocarcinoma transcript 1 (MALAT1) results in the decrease of E-cadherin expression and the increased expression of mesenchymal markers leading to enhanced EMT [49]. MALAT1 is a prognostic marker in several cancers: including lung, breast, pancreas, liver, colon, uterus, cervix, and prostate [50,51]. In renal cancer, reciprocal crosstalk among MALAT1, miR205, and EZH2 suppresses the expression of E-Cadherin and enhances Wnt signaling to promote cancer metastasis [52] (Table 1).

#### 4.2.3. lncRNA-ZEB2NAT

ZEB2 Natural antisense transcript regulates Zeb2/Sip1 gene expression during Snail1-induced EMT. While Snail1 does not affect the synthesis of the Zeb2 mRNA, it prevents the processing of a specific intron in the 5′- UTR. This intron contains an internal ribosome entry site (IRES) essential for the expression of Zeb2. The maintenance of this intron is dependent on the expression of ZEB2NAT which overlaps with the 5′ splice site [53]. TGFβ1, secreted by cancer-associated fibroblasts, induces an epithelial-mesenchymal transition of bladder cancer cells in a mechanism dependent on ZEB2NAT expression [54] (Table 1).

#### 4.2.4. HOTAIR

HOX antisense intergenic RNA (HOTAIR), a lncRNA encoded by a gene located in the mammalian HOXC locus, binds to the polycomb repressive complex 2 (PRC2), a histone methyltransferase required for epigenetic silencing during development and cancer [55,56,57]. HOTAIR is overexpressed in both NSCLC and breast cancer and its expression level correlates with poor disease outcome [55,58] (Table 1). HOTAIR upregulation was also observed in several other cancer types where numerous regulatory factors control its expression, including TGF-β. Overall, HOTAIR is involved in multiple processes such as mobility, proliferation, apoptosis and invasion of tumor cells, and is therefore particularly relevant in EMT context. The silencing of HOTAIR in colon cancer cells is followed by the concomitant increase in E-cadherin expression and decrease in vimentin expression, demonstrating the direct link between HOTAIR and EMT [59]. Importantly, it was demonstrated that the control of EMT through HOTAIR contributes to the emergence and maintenance of cancer stem cells (CSCs) [60].

#### 4.2.5. lncRNA-HIT

The HOXA transcript induced by TGF-β (lncRNA-HIT) contributes to EMT in breast carcinoma cells, and its elevated expression is associated with invasion-prone human primary breast carcinoma cells [61] (Table 1).

#### 4.2.6. MEG3

Maternally-expressed 3 (MEG3) forms RNA-DNA triplex structures with genes involved in the TGF-β pathway and ultimately modulates tissue invasion in breast and lung cancer cells [62,63] (Table 1). MEG3 is expressed at significantly lower levels in invasive ductal carcinoma, as well as in the aggressive and difficult-to-treat basal molecular subtype, as compared to normal breast tissue. Finally, tumors maintaining low MEG3 expression feature higher levels of TGF-β-associated genes such as TGFB2, TGFBR1, and SMAD2 [62].

### 4.3. Other Non-Coding RNA Species

#### 4.3.1. Circular RNAs

Circular RNAs (circRNAs) are expressed in mammalian cells and form a covalently-closed continuous loop. CirRNAs function mainly as sponges for miRNAs and RNA-binding proteins (RBP) [64]. Reports on differential expressions of circular RNAs between cancerous and non-cancerous samples are emerging in the literature suggesting their implication in tumor progression [65,66,67,68,69]. In breast epithelial HMLE cells experiencing TGF-β-induced EMT, the expression of hundreds of circRNAs fluctuates. Under these circumstances, the production of over one-third of the most abundant circRNAs are regulated by the Quaking alternative splicing factor [70]. 

#### 4.3.2. PIWI-interacting RNAs

Originally observed in the germline, PIWI-interacting RNAs (piRNAs) are small non-coding RNAs which recently emerged as potential markers in tumor progression [71,72]. With over 20,000 genes, piRNA-encoding genes are highly represented in the human genome. piRNAs function primarily in the nucleus and interact with members of the Argonaute family, such as PIWI proteins [71]. Aberrant patterns of piRNA expression, and the dysregulation of key enzymes involved in their biogenesis, are frequently observed in diverse tumor types, such as breast and lung cancers [72,73]. In particular, the PIWI-like RNA-mediated gene silencing 2 (PIWIL2), a member of the Argonaute family involved in piRNA processing, is significantly overexpressed in both breast cancer stem cells and TGF-β-induced EMT [74].

#### 4.3.3. Small Nucleolar and Small Nuclear RNAs

Small nucleolar RNAs (snoRNAs) and small nuclear RNAs (snRNAs) are 60 to 300 nucleotides long. SnoRNAs are involved in the post-transcriptional modification of ribosomal RNA and play integral roles in the formation of small nucleolar ribonucleoprotein particles (snoRNP). SnRNAs support RNA-RNA interactions and spliceosome assembly [75]. Dysregulation of snoRNA and/or snRNA expression is commonly observed in cancers. Several snoRNAs are upregulated in murine and human breast cancer as well as in prostate cancers; interfering with their biogenesis suppresses cell growth and colony formation in MCF-7, U-20S, and A549 cells in vitro, and ultimately compromises tumorigenicity in vivo [76]. Although the exact contribution of small nuclear RNAs (snRNAs) in tumor progression and metastasis is not yet clearly established, the 7SK snRNA indirectly participates in the control of EMT (Table 1). 7SK snRNAs form dynamic complexes with the La-related protein LARP7. These complexes often involve additional molecular partners, such as positive transcription elongation factor b (P-TEFb), whose activity is relevant to EMT-related tumor progression in breast tissue. Decreased levels of LARP7 and 7SK snRNA redistribute P-TEFb toward the transcriptionally active super elongation complex (SEC), resulting in P-TEFb activation and increased transcription of EMT transcription factors including Slug, FOXC2, ZEB2, and Twist1, ultimately promoting EMT, invasion, and metastasis of breast tumor cells [77].

#### 4.3.4. Transfer RNAs

tRNA are abundant molecules which represent 30% of the total RNA pool in eukaryotic cells [78]. The human genome contains over 600 tRNA genes, which are scattered throughout the genome and are present on all but the Y chromosome [79]. Until recently, tRNAs have been considered to be housekeeping molecules dedicated to protein translation. However, a growing body of evidence indicates that differential tRNA expression deeply influences the whole dynamic of translation, supporting or impairing the expression of particular proteins [80,81,82,83].

The phenotypic changes associated with EMT and tumor progression imply a radical reprogramming of the proteome and a significant redistribution of the overall codon usage. Considering tRNA molecules in translation as the ‘supply’ and codon usage as the ‘demand’, it has been proposed that the codon demand associated with EMT and tumorigenesis is mediated by a transcriptional regulation of the tRNA supply [84,85]. Moreover, the role of tRNAs in the regulation of gene expression was recently supported in a recent study based on the analysis of tRNA content in various human cell lines and tissue samples [85]. It was observed that distinct tRNA signatures correlate with either proliferation or differentiation, two distinct cellular program often described for their role during tumor progression [85,86,87,88]. In cancer-derived breast cell lines overall tRNA levels are upregulated by up to three-fold compared to non-cancer control cells [81]. A selective upregulation of tRNA^Glu^_UUC_ and tRNA^Arg^_CCG_ was also observed during breast tumor progression, and has been proposed to contribute to metastatic progression of breast tumor cells through the enhanced translation of pro-metastatic transcripts enriched in the corresponding codons [80] (Table 1).

Transfer RNA-derived RNA fragments (tRFs) were also documented for their role in tumor progression. tRFs belong to a family of short non-coding RNAs that are constitutively expressed or activated upon specific growth conditions. This tRNA subspecies are presumably generated through the processing by Dicer and RNAse Z [89,90] or through the action of specific ribonucleases for the stress-induced tRFs fragments (tiRNAs) [91,92]. These small RNAs have distinct functions in various biological processes, including tumor suppression and protein regulation [93]. tRNA fragments have also been reported to promote cell migration as illustrated by miR-720 in cervical cancer cells in vitro [94]. Additionally, in a study on the epithelialization of mouse ovarian tumor cells in Dicer-Pten double-knock-out mice, tRNA overexpression was identified in tumors in the context of a miRNA profiling experiment, and was associated to oncogenic transformation [95].

While tRNA expression and enhanced translation capacity appear relevant in tumor progression of carcinomas, their regulation and contribution during TGF-β-induced EMT have not been documented yet. With the intention to further the discussion on the involvement of tRNAs in EMT, our laboratories developed a strategy, based on metabolic RNA labeling followed by microarray analysis, to evaluate global tRNA changes upon TGF-β treatment in tumor cells in vitro. The corresponding original experimental evidence is described and examined in the following section.

## 5. Evidence of Selective Regulation of tRNA Expression during TGF-β-Induced EMT

**Figure 3:**
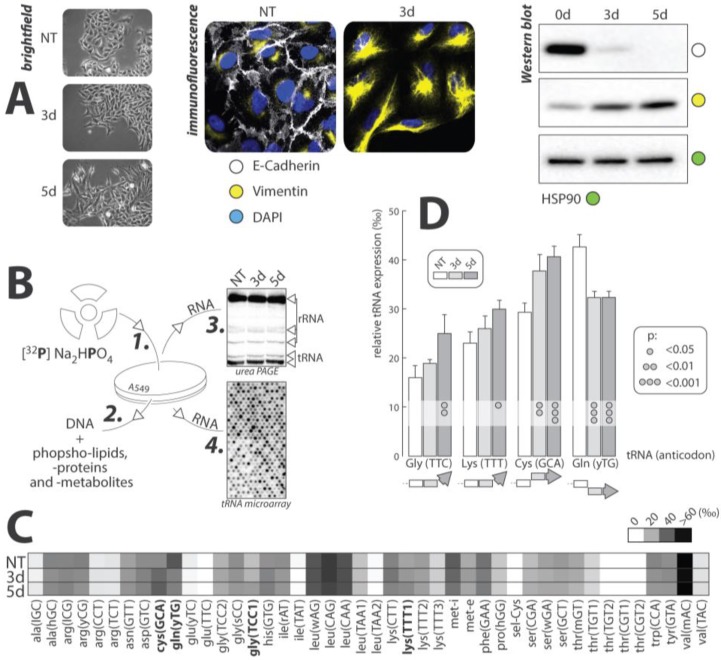
(**A**) Cell morphology and expression of EMT markers**:** A549 cells pictured after no-treatment (NT), three days of TGFβ treatment (3d), and 5d of TGFβ treatment. Cells begin with a slightly mesenchymal phenotype in the NT, but display a completely mesenchymal phenotype after prolonged exposure to TGFβ. E-cadherin (white) is the epithelial cell marker and vimentin (yellow) is the mesenchymal cell marker. DAPI (blue) is the counterstain used to stain nuclei. HSP90 was used as a loading control. (**B**) Overview of the experimental Procedure: **1**. Radioactive orthophosphate was added to cell cultures at onset of experiment. **2**. Total RNAs were Trizol extracted and all other labeled molecules were removed from sample. **3**. Labeling of tRNA molecules was confirmed via gel electrophoresis. **4**. Samples were hybridized to tRNA microarrays and analyzed. (**C**) Average tRNA expression: The heat map shows the average number of each tRNA (per thousand) that is present across the three conditions. tRNA abundances range from close to 0 to over 60‰. (**D**) Statistically significant results: The relative tRNA expression, per thousand, of the four tRNA that displayed significant changes in tRNA expression throughout EMT are shown along with standard error bars. Both 3d and 5d were compared to the NT and the statistical significance of those changes can be seen from the dot on the bars. The overall trend of expression for each tRNA is shown below the graph.

The effects of TGFβ-mediated EMT on the expression of tRNAs was examined in three experimental conditions: no treatment (NT), three days of TGFβ exposure (3d), and five days (5d) of TGFβ exposure. This experiment was carried out on the human lung cancer cell line A549. These cells are a common model used in TGFβ mediated EMT, as they respond remarkably to TGFβ treatment. Cells were grown in media containing radioactive orthophosphate (^32^P), which non-specifically labeled RNA, DNA, phosphoproteins, and other phosphate-containing metabolites. Total RNAs were extracted and hybridized to tRNA microarrays for analysis according to published protocols. Extracted RNAs were also analyzed by gel electrophoresis, to ensure that tRNAs were effectively radiolabeled (Figure 3).

EMT progression was confirmed visually by immunofluorescent microscopy and Western blot. The epithelial cell marker, E-cadherin, and the mesenchymal cell marker, vimentin, were targeted to track the transition. In both experiments, levels of E-cadherin were greatest in the NT culture, and decreased rapidly upon addition of TGFβ. The opposite was true for the expression of vimentin, which increased with prolonged exposure to TGFβ. These data offered positive identification of EMT progression in our cell model.

tRNA expression within each of the experimental cultures was measured using microarray analysis according to published protocols [96]. Array data were collected in triplicate and averaged to generate a heat map of overall tRNA expression throughout EMT (Figure 3). Seleno-cysteine (sel-Cys) represents the least expressed tRNA and valine (val) represents the tRNA with the greatest overall expression in A549 cells. Four tRNAs, including cysteine (GCA), glutamine (yTG), glycine (TCC), and lysine (TTT), displayed statistically significant changes in expression throughout the transition. The relative tRNA expression throughout the three experimental cell cultures for these four tRNA was plotted and standard error bars, as well as statistical significance, was shown (Figure 3).

Between the four significant tRNAs, the trends of expression change were not consistent, with only lysine and cysteine showing a similar trend of increasing between NT and 3d, and showing no significant change between the 3d and 5d. Glycine also showed an increasing trend, only changing significantly after five days of treatment with TGFβ. Glutamine, however, displayed a decreasing trend, with both the 3d and 5d cells showed a significantly lower amount of this tRNA than in the NT cells.

There are no commonalities between our results and the type of amino acid carried by the tRNA. Cysteine and glutamine have polar, uncharged side-chains, whereas glycine has a non-polar, aliphatic side-chain, and lysine’s side-chain is positively charged. The significance of these tRNA expression patterns with respect to codon usage within proteins in EMT has yet to be explored. Another possible explanation for the change in the expression of these four tRNAs could be an increase in non-translational tRNA activity, such as the production of tRFs as described in the above section.

Though the role of the change in expression of these tRNAs has not been specifically identified at this point, there is the possibility that tRNA may play a role in the progression of EMT, in its regulation, or perhaps could be utilized as EMT markers in the future. Overall non-coding RNAs whose expression fluctuates during EMT deserve special attention as they represent a reservoir of targets and offer potential therapeutic opportunities to prevent EMT-associated metastasis. Fluctuations in abundant and supposedly housekeeping species, such as tRNAs, emphasize the necessity to cast a broad net in the current quest of new regulatory RNAs.

## Figures and Tables

**Table 1:** Non-Coding RNA involved in EMT.

Non-Coding RNA	Relevant Examples	Specific Function	Most Described Targets	Related Cancers	References
**miRNAs**	miR-1 * miR-200 family * miR-205 * miR-203 *	Epithelial maintenance	ZEB1/2↓ Slug↓ Bmi1↓	Breast Lung Prostate	[5,35,37,39,40]
**LncRNAs**	LncRNA-ATB ^†^ MALAT1 ^†^ lncRNA-ZEB2NAT ^†^ HOTAIR ^†^ lncRNA-HIT	Tumor cell invasion; Organ colonization; Proliferation; Cancer Stem Cells	ZEB1/2↑ IL-11↑ miR-200↓ miR-205↓ E-cadherin↓	Prostate Lung Breast Kidney Pancreas Liver Colon Uterus	[44,45,46,47,49,51,52,53,54,55,57,58,59,61]
	MEG3 *	TGF-β pathway regulation	TGFBR1↑ TGFB2↑ SMAD2↑	Breast	[62,63]
**circRNAs**	CDR1as/ciRS-7 *	miRNA sponge	miRNA-7↓	Colon	[66,67,69]
**piRNAs**	Pir-932 ^†^	Stemness properties	Latexin↓	Breast	[74]
**snoRNAs** **snRNAs**	7SK snRNA *	Tumor cell invasion	Slug↓ FOXC2↓ ZEB2↓ Twist1↓	Breast	[77]
**tRNAs**	tRNA^Glu^_UUC_^†^	Tumor progression	EXOSC2↓ GRIPAP1↓	Breast	[80]
	MicroRNA-720 ^†^	Tumor cell motility	Rab35↓	Uterus	[94]

* Epithelial non-coding RNAs; ^†^ Mesenchymal non-coding RNAs.

## References

[1] Nieto M.A., Huang R.Y.-J., Jackson R.A., Thiery J.P. (2016). EMT: 2016. Cell.

[2] Thiery J.P., Acloque H., Huang R.Y.J., Nieto M.A. (2009). Epithelial-mesenchymal transitions in development and disease. Cell.

[3] Zheng X., Carstens J.L., Kim J., Scheible M., Kaye J., Sugimoto H., Wu C.-C., LeBleu V.S., Kalluri R. (2015). Epithelial-to-mesenchymal transition is dispensable for metastasis but induces chemoresistance in pancreatic cancer. Nature.

[4] Fischer K.R., Durrans A., Lee S., Sheng J., Li F., Wong S.T.C., Choi H., El Rayes T., Ryu S., Troeger J. (2015). Epithelial-to-mesenchymal transition is not required for lung metastasis but contributes to chemoresistance. Nature.

[5] Krebs A.M., Mitschke J., Lasierra Losada M., Schmalhofer O., Boerries M., Busch H., Boettcher M., Mougiakakos D., Reichardt W., Bronsert P. (2017). The EMT-activator Zeb1 is a key factor for cell plasticity and promotes metastasis in pancreatic cancer. Nat. Cell Biol..

[6] Francart M.-E., Lambert J., Vanwynsberghe A.M., Thompson E.W., Bourcy M., Polette M., Gilles C. (2017). Epithelial-Mesenchymal Plasticity and Circulating Tumor Cells: Travel Companions to Metastases. Dev. Dyn. Off. Publ. Am. Assoc. Anat..

[7] Kalluri R., Weinberg R.A. (2009). The basics of epithelial-mesenchymal transition. J. Clin. Investig..

[8] Smith B.N., Bhowmick N.A. (2016). Role of EMT in Metastasis and Therapy Resistance. J. Clin. Med..

[9] Schedin P., Borges V. (2009). Breaking down barriers: the importance of the stromal microenvironment in acquiring invasiveness in young women’s breast cancer. Breast Cancer Res. BCR.

[10] Kalluri R., Zeisberg M. (2006). Fibroblasts in cancer. Nat. Rev. Cancer.

[11] Lamouille S., Xu J., Derynck R. (2014). Molecular mechanisms of epithelial–mesenchymal transition. Nat. Rev. Mol. Cell Biol..

[12] Massagué J. (2008). TGFβ in Cancer. Cell.

[13] Bourcy M., Suarez-Carmona M., Lambert J., Francart M.-E., Schroeder H., Delierneux C., Skrypek N., Thompson E.W., Jérusalem G., Berx G. (2016). Tissue Factor Induced by Epithelial-Mesenchymal Transition Triggers a Procoagulant State That Drives Metastasis of Circulating Tumor Cells. Cancer Res..

[14] Derynck R., Zhang Y.E. (2003). Smad-dependent and Smad-independent pathways in TGF-β family signalling. Nature.

[15] Valcourt U., Kowanetz M., Niimi H., Heldin C.-H., Moustakas A. (2005). TGF-beta and the Smad signaling pathway support transcriptomic reprogramming during epithelial-mesenchymal cell transition. Mol. Biol. Cell.

[16] Zhang H., Liu L., Wang Y., Zhao G., Xie R., Liu C., Xiao X., Wu K., Nie Y., Zhang H. (2013). KLF8 involves in TGF-beta-induced EMT and promotes invasion and migration in gastric cancer cells. J. Cancer Res. Clin. Oncol..

[17] Larocca C., Cohen J.R., Fernando R.I., Huang B., Hamilton D.H., Palena C. (2013). An autocrine loop between TGF-β1 and the transcription factor Brachyury controls the transition of human carcinoma cells into a mesenchymal phenotype. Mol. Cancer Ther..

[18] Hardin H., Guo Z., Shan W., Montemayor-Garcia C., Asioli S., Yu X.-M., Harrison A.D., Chen H., Lloyd R.V. (2014). The Roles of the Epithelial-Mesenchymal Transition Marker PRRX1 and miR-146b-5p in Papillary Thyroid Carcinoma Progression. Am. J. Pathol..

[19] Batlle E., Sancho E., Francí C., Domínguez D., Monfar M., Baulida J., García De Herreros A. (2000). The transcription factor snail is a repressor of E-cadherin gene expression in epithelial tumour cells. Nat. Cell Biol..

[20] Cano A., Pérez-Moreno M.A., Rodrigo I., Locascio A., Blanco M.J., del Barrio M.G., Portillo F., Nieto M.A. (2000). The transcription factor snail controls epithelial-mesenchymal transitions by repressing E-cadherin expression. Nat. Cell Biol..

[21] Xiang J., Fu X., Ran W., Wang Z. (2017). Grhl2 reduces invasion and migration through inhibition of TGFβ-induced EMT in gastric cancer. Oncogenesis.

[22] Yao B., Zhao J., Li Y., Li H., Hu Z., Pan P., Zhang Y., Du E., Liu R., Xu Y. (2015). Elf5 inhibits TGF-β-driven epithelial-mesenchymal transition in prostate cancer by repressing SMAD3 activation. Prostate.

[23] Cardenas H., Vieth E., Lee J., Segar M., Liu Y., Nephew K.P., Matei D. (2014). TGF-β induces global changes in DNA methylation during the epithelial-to-mesenchymal transition in ovarian cancer cells. Epigenetics.

[24] Serrano-Gomez S.J., Maziveyi M., Alahari S.K. (2016). Regulation of epithelial-mesenchymal transition through epigenetic and post-translational modifications. Mol. Cancer.

[25] Roche J., Nasarre P., Gemmill R., Baldys A., Pontis J., Korch C., Guilhot J., Ait-Si-Ali S., Drabkin H. (2013). Global Decrease of Histone H3K27 Acetylation in ZEB1-Induced Epithelial to Mesenchymal Transition in Lung Cancer Cells. Cancers.

[26] Bedi U., Mishra V.K., Wasilewski D., Scheel C., Johnsen S.A. (2014). Epigenetic plasticity: A central regulator of epithelial-to-mesenchymal transition in cancer. Oncotarget.

[27] De Craene B., Berx G. (2013). Regulatory networks defining EMT during cancer initiation and progression. Nat. Rev. Cancer.

[28] Evdokimova V., Tognon C., Ng T., Ruzanov P., Melnyk N., Fink D., Sorokin A., Ovchinnikov L.P., Davicioni E., Triche T.J. (2009). Translational activation of snail1 and other developmentally regulated transcription factors by YB-1 promotes an epithelial-mesenchymal transition. Cancer Cell.

[29] Chaudhury A., Hussey G.S., Ray P.S., Jin G., Fox P.L., Howe P.H. (2010). TGF-beta-mediated phosphorylation of hnRNP E1 induces EMT via transcript-selective translational induction of Dab2 and ILEI. Nat. Cell Biol..

[30] Hussey G.S., Chaudhury A., Dawson A.E., Lindner D.J., Knudsen C.R., Wilce M.C.J., Merrick W.C., Howe P.H. (2011). Identification of an mRNP Complex Regulating Tumorigenesis at the Translational Elongation Step. Mol. Cell.

[31] Grelet S., Andries V., Polette M., Gilles C., Staes K., Martin A.-P., Kileztky C., Terryn C., Dalstein V., Cheng C.-W. (2015). The human NANOS3 gene contributes to lung tumour invasion by inducing epithelial-mesenchymal transition. J. Pathol..

[32] Hussey G.S., Link L.A., Brown A.S., Howley B.V., Chaudhury A., Howe P.H. (2012). Establishment of a TGFβ-Induced Post-Transcriptional EMT Gene Signature. PLOS ONE.

[33] Bartel D.P. (2009). MicroRNAs: Target recognition and regulatory functions. Cell.

[34] Londin E., Loher P., Telonis A.G., Quann K., Clark P., Jing Y., Hatzimichael E., Kirino Y., Honda S., Lally M. (2015). Analysis of 13 cell types reveals evidence for the expression of numerous novel primate- and tissue-specific microRNAs. Proc. Natl. Acad. Sci. USA.

[35] Gregory P.A., Bert A.G., Paterson E.L., Barry S.C., Tsykin A., Farshid G., Vadas M.A., Khew-Goodall Y., Goodall G.J. (2008). The miR-200 family and miR-205 regulate epithelial to mesenchymal transition by targeting ZEB1 and SIP1. Nat. Cell Biol..

[36] Korpal M., Lee E.S., Hu G., Kang Y. (2008). The miR-200 Family Inhibits Epithelial-Mesenchymal Transition and Cancer Cell Migration by Direct Targeting of E-cadherin Transcriptional Repressors ZEB1 and ZEB2. J. Biol. Chem..

[37] Brabletz S., Brabletz T. (2010). The ZEB/miR-200 feedback loop—A motor of cellular plasticity in development and cancer?. EMBO Rep..

[38] Gregory P.A., Bracken C.P., Smith E., Bert A.G., Wright J.A., Roslan S., Morris M., Wyatt L., Farshid G., Lim Y.-Y. (2011). An autocrine TGF-β/ZEB/miR-200 signaling network regulates establishment and maintenance of epithelial-mesenchymal transition. Mol. Biol. Cell.

[39] Ding X., Park S.I., McCauley L.K., Wang C.-Y. (2013). Signaling between Transforming Growth Factor β (TGF-β) and Transcription Factor SNAI2 Represses Expression of MicroRNA miR-203 to Promote Epithelial-Mesenchymal Transition and Tumor Metastasis. J. Biol. Chem..

[40] Wellner U., Schubert J., Burk U.C., Schmalhofer O., Zhu F., Sonntag A., Waldvogel B., Vannier C., Darling D., zur Hausen A. (2009). The EMT-activator ZEB1 promotes tumorigenicity by repressing stemness-inhibiting microRNAs. Nat. Cell Biol..

[41] Liu Y.-N., Yin J.J., Abou-Kheir W., Hynes P.G., Casey O.M., Fang L., Yi M., Stephens R.M., Seng V., Sheppard-Tillman H. (2013). MiR-1 and miR-200 inhibit EMT via Slug-dependent and tumorigenesis via Slug-independent mechanisms. Oncogene.

[42] Meseure D., Drak Alsibai K., Nicolas A., Bieche I., Morillon A. (2015). Long Noncoding RNAs as New Architects in Cancer Epigenetics, Prognostic Biomarkers, and Potential Therapeutic Targets. BioMed Res. Int..

[43] Tay Y., Rinn J., Pandolfi P.P. (2014). The multilayered complexity of ceRNA crosstalk and competition. Nature.

[44] Yuan J., Yang F., Wang F., Ma J., Guo Y., Tao Q., Liu F., Pan W., Wang T., Zhou C. (2014). A Long Noncoding RNA Activated by TGF-β Promotes the Invasion-Metastasis Cascade in Hepatocellular Carcinoma. Cancer Cell.

[45] Xu S., Yi X.-M., Tang C.-P., Ge J.-P., Zhang Z.-Y., Zhou W.-Q. (2016). Long non-coding RNA ATB promotes growth and epithelial-mesenchymal transition and predicts poor prognosis in human prostate carcinoma. Oncol. Rep..

[46] Iguchi T., Uchi R., Nambara S., Saito T., Komatsu H., Hirata H., Ueda M., Sakimura S., Takano Y., Kurashige J. (2015). A long noncoding RNA, lncRNA-ATB, is involved in the progression and prognosis of colorectal cancer. Anticancer Res..

[47] Ke L., Xu S.-B., Wang J., Jiang X.-L., Xu M.-Q. (2017). High expression of long non-coding RNA ATB indicates a poor prognosis and regulates cell proliferation and metastasis in non-small cell lung cancer. Clin. Transl. Oncol..

[48] Shi S.-J., Wang L.-J., Yu B., Li Y.-H., Jin Y., Bai X.-Z. (2015). LncRNA-ATB promotes trastuzumab resistance and invasion-metastasis cascade in breast cancer. Oncotarget.

[49] Fan Y., Shen B., Tan M., Mu X., Qin Y., Zhang F., Liu Y. (2014). TGF-β-induced upregulation of malat1 promotes bladder cancer metastasis by associating with suz12. Clin. Cancer Res..

[50] Shi X., Sun M., Liu H., Yao Y., Song Y. (2013). Long non-coding RNAs: A new frontier in the study of human diseases. Cancer Lett..

[51] Ji P., Diederichs S., Wang W., Böing S., Metzger R., Schneider P.M., Tidow N., Brandt B., Buerger H., Bulk E. (2003). MALAT-1, a novel noncoding RNA, and thymosin beta4 predict metastasis and survival in early-stage non-small cell lung cancer. Oncogene.

[52] Hirata H., Hinoda Y., Shahryari V., Deng G., Nakajima K., Tabatabai Z.L., Ishii N., Dahiya R. (2015). Long Noncoding RNA MALAT1 Promotes Aggressive Renal Cell Carcinoma through Ezh2 and Interacts with miR-205. Cancer Res..

[53] Beltran M., Puig I., Peña C., García J.M., Álvarez A.B., Peña R., Bonilla F., de Herreros A.G. (2008). A natural antisense transcript regulates Zeb2/Sip1 gene expression during Snail1-induced epithelial–mesenchymal transition. Genes Dev..

[54] Zhuang J., Lu Q., Shen B., Huang X., Shen L., Zheng X., Huang R., Yan J., Guo H. (2015). TGFβ1 secreted by cancer-associated fibroblasts induces epithelial-mesenchymal transition of bladder cancer cells through lncRNA-ZEB2NAT. Sci. Rep..

[55] Gupta R.A., Shah N., Wang K.C., Kim J., Horlings H.M., Wong D.J., Tsai M.-C., Hung T., Argani P., Rinn J.L. (2010). Long non-coding RNA HOTAIR reprograms chromatin state to promote cancer metastasis. Nature.

[56] Davidovich C., Zheng L., Goodrich K.J., Cech T.R. (2013). Promiscuous RNA binding by Polycomb Repressive Complex 2. Nat. Struct. Mol. Biol..

[57] Rinn J.L., Kertesz M., Wang J.K., Squazzo S.L., Xu X., Brugmann S.A., Goodnough L.H., Helms J.A., Farnham P.J., Segal E. (2007). Functional demarcation of active and silent chromatin domains in human HOX loci by noncoding RNAs. Cell.

[58] Nakagawa T., Endo H., Yokoyama M., Abe J., Tamai K., Tanaka N., Sato I., Takahashi S., Kondo T., Satoh K. (2013). Large noncoding RNA HOTAIR enhances aggressive biological behavior and is associated with short disease-free survival in human non-small cell lung cancer. Biochem. Biophys. Res. Commun..

[59] Wu Z.-H., Wang X.-L., Tang H.-M., Jiang T., Chen J., Lu S., Qiu G.-Q., Peng Z.-H., Yan D.-W. (2014). Long non-coding RNA HOTAIR is a powerful predictor of metastasis and poor prognosis and is associated with epithelial-mesenchymal transition in colon cancer. Oncol. Rep..

[60] Hajjari M., Khoshnevisan A., Shin Y.K. (2014). Molecular function and regulation of long non-coding RNAs: paradigms with potential roles in cancer. Tumour Biol..

[61] Richards E.J., Zhang G., Li Z.-P., Permuth-Wey J., Challa S., Li Y., Kong W., Dan S., Bui M.M., Coppola D., Mao W.-M. (2015). Long non-coding RNAs (LncRNA) regulated by transforming growth factor (TGF) β: LncRNA-hit-mediated TGFβ-induced epithelial to mesenchymal transition in mammary epithelia. J. Biol. Chem..

[62] Mondal T., Subhash S., Vaid R., Enroth S., Uday S., Reinius B., Mitra S., Mohammed A., James A.R., Hoberg E. (2015). MEG3 long noncoding RNA regulates the TGF-β pathway genes through formation of RNA–DNA triplex structures. Nat. Commun..

[63] Terashima M., Tange S., Ishimura A., Suzuki T. (2016). MEG3 long noncoding RNA contributes to the epigenetic regulation of epithelial-mesenchymal transition in lung cancer cell lines. J. Biol. Chem..

[64] Dong Y., He D., Peng Z., Peng W., Shi W., Wang J., Li B., Zhang C., Duan C. (2017). Circular RNAs in cancer: an emerging key player. J. Hematol. Oncol..

[65] Bachmayr-Heyda A., Reiner A.T., Auer K., Sukhbaatar N., Aust S., Bachleitner-Hofmann T., Mesteri I., Grunt T.W., Zeillinger R., Pils D. (2015). Correlation of circular RNA abundance with proliferation—Exemplified with colorectal and ovarian cancer, idiopathic lung fibrosis, and normal human tissues. Sci. Rep..

[66] Hansen T.B., Kjems J., Damgaard C.K. (2013). Circular RNA and miR-7 in cancer. Cancer Res..

[67] Li J., Yang J., Zhou P., Le Y., Zhou C., Wang S., Xu D., Lin H.-K., Gong Z. (2015). Circular RNAs in cancer: novel insights into origins, properties, functions and implications. Am. J. Cancer Res..

[68] Hansen T.B., Jensen T.I., Clausen B.H., Bramsen J.B., Finsen B., Damgaard C.K., Kjems J. (2013). Natural RNA circles function as efficient microRNA sponges. Nature.

[69] Weng W., Wei Q., Toden S., Yoshida K., Nagasaka T., Fujiwara T., Cai S., Qin H., Ma Y., Goel A. (2017). Circular RNA ciRS-7—A promising prognostic biomarker and a potential therapeutic target in colorectal cancer. Clin. Cancer Res..

[70] Conn S.J., Pillman K.A., Toubia J., Conn V.M., Salmanidis M., Phillips C.A., Roslan S., Schreiber A.W., Gregory P.A., Goodall G.J. (2015). The RNA Binding Protein Quaking Regulates Formation of circRNAs. Cell.

[71] Ng K.W., Anderson C., Marshall E.A., Minatel B.C., Enfield K.S.S., Saprunoff H.L., Lam W.L., Martinez V.D. (2016). Piwi-interacting RNAs in cancer: Emerging functions and clinical utility. Mol. Cancer.

[72] Hashim A., Rizzo F., Marchese G., Ravo M., Tarallo R., Nassa G., Giurato G., Santamaria G., Cordella A., Cantarella C. (2014). RNA sequencing identifies specific PIWI-interacting small non-coding RNA expression patterns in breast cancer. Oncotarget.

[73] Huang G., Hu H., Xue X., Shen S., Gao E., Guo G., Shen X., Zhang X. (2013). Altered expression of piRNAs and their relation with clinicopathologic features of breast cancer. Clin. Transl. Oncol..

[74] Zhang H., Ren Y., Xu H., Pang D., Duan C., Liu C. (2013). The expression of stem cell protein Piwil2 and piR-932 in breast cancer. Surg. Oncol..

[75] Rapisuwon S., Vietsch E.E., Wellstein A. (2016). Circulating biomarkers to monitor cancer progression and treatment. Comput. Struct. Biotechnol. J..

[76] Su H., Xu T., Ganapathy S., Shadfan M., Long M., Huang T.H.-M., Thompson I., Yuan Z.-M. (2014). Elevated snoRNA biogenesis is essential in breast cancer. Oncogene.

[77] Ji X., Lu H., Zhou Q., Luo K. (2014). LARP7 suppresses P-TEFb activity to inhibit breast cancer progression and metastasis. Elife.

[78] Waldron C., Lacroute F. (1975). Effect of growth rate on the amounts of ribosomal and transfer ribonucleic acids in yeast. J. Bacteriol..

[79] Goodenbour J.M., Pan T. (2006). Diversity of tRNA genes in eukaryotes. Nucleic Acids Res..

[80] Goodarzi H., Nguyen H.C.B., Zhang S., Dill B.D., Molina H., Tavazoie S.F. (2016). Modulated Expression of Specific tRNAs Drives Gene Expression and Cancer Progression. Cell.

[81] Pavon-Eternod M., Gomes S., Geslain R., Dai Q., Rosner M.R., Pan T. (2009). tRNA over-expression in breast cancer and functional consequences. Nucleic Acids Res..

[82] Rudolph K.L.M., Schmitt B.M., Villar D., White R.J., Marioni J.C., Kutter C., Odom D.T. (2016). Codon-Driven Translational Efficiency Is Stable across Diverse Mammalian Cell States. PLOS Genet..

[83] Geslain R., Eriani G. (2014). Regulation of translation dynamic and neoplastic conversion by tRNA and their pieces. Transl. Austin..

[84] Gingold H., Pilpel Y. (2011). Determinants of translation efficiency and accuracy. Mol. Syst. Biol..

[85] Gingold H., Tehler D., Christoffersen N.R., Nielsen M.M., Asmar F., Kooistra S.M., Christophersen N.S., Christensen L.L., Borre M., Sørensen K.D. (2014). A dual program for translation regulation in cellular proliferation and differentiation. Cell.

[86] Ruijtenberg S., van den Heuvel S. (2016). Coordinating cell proliferation and differentiation: Antagonism between cell cycle regulators and cell type-specific gene expression. Cell Cycle.

[87] Kumar S.M., Liu S., Lu H., Zhang H., Zhang P.J., Gimotty P.A., Guerra M., Guo W., Xu X. (2012). Acquired cancer stem cell phenotypes through Oct4-mediated dedifferentiation. Oncogene.

[88] Klochendler A., Weinberg-Corem N., Moran M., Swisa A., Pochet N., Savova V., Vikeså J., Van de Peer Y., Brandeis M., Regev A. (2012). A Transgenic Mouse Marking Live Replicating Cells Reveals In Vivo Transcriptional Program of Proliferation. Dev. Cell.

[89] Cole C., Sobala A., Lu C., Thatcher S.R., Bowman A., Brown J.W.S., Green P.J., Barton G.J., Hutvagner G. (2009). Filtering of deep sequencing data reveals the existence of abundant Dicer-dependent small RNAs derived from tRNAs. RNA NY.

[90] Lee Y.S., Shibata Y., Malhotra A., Dutta A. (2009). A novel class of small RNAs: tRNA-derived RNA fragments (tRFs). Genes Dev..

[91] Saikia M., Jobava R., Parisien M., Putnam A., Krokowski D., Gao X.-H., Guan B.-J., Yuan Y., Jankowsky E., Feng Z. (2014). Angiogenin-cleaved tRNA halves interact with cytochrome c, protecting cells from apoptosis during osmotic stress. Mol. Cell. Biol..

[92] Fu H., Feng J., Liu Q., Sun F., Tie Y., Zhu J., Xing R., Sun Z., Zheng X. (2009). Stress induces tRNA cleavage by angiogenin in mammalian cells. FEBS Lett..

[93] Kumar P., Kuscu C., Dutta A. (2016). Biogenesis and Function of Transfer RNA-Related Fragments (tRFs). Trends Biochem. Sci..

[94] Tang Y., Lin Y., Li C., Hu X., Liu Y., He M., Luo J., Sun G., Wang T., Li W. (2015). MicroRNA-720 promotes in vitro cell migration by targeting Rab35 expression in cervical cancer cells. Cell Biosci..

[95] Hua Y., Choi P.-W., Trachtenberg A.J., Ng A.C., Kuo W.P., Ng S.-K., Dinulescu D.M., Matzuk M.M., Berkowitz R.S., Ng S.-W. (2016). Epithelialization of mouse ovarian tumor cells originating in the fallopian tube stroma. Oncotarget.

[96] Grelet S., McShane A., Hok E., Tomberlin J., Howe P.H., Geslain R. (2017). SPOt: A novel and streamlined microarray platform for observing cellular tRNA levels. PLOS ONE.

